# Influence of an Axial-Electromagnetic Field Treatment Device with a Solenoid Structure on Crystallization Fouling on the Tube Side of a Shell-and-Tube Heat Exchanger

**DOI:** 10.3390/e25070962

**Published:** 2023-06-21

**Authors:** Yaxuan Peng, Xuefei Xu, Yandong Liang

**Affiliations:** 1 School of Civil Engineering and Architecture, Northeast Electric Power University, Jilin 132012, China; pengyx_neepu@sina.com; 2 School of Automation Engineering, Northeast Electric Power University, Jilin 132012, China; xuxuefei2022@163.com

**Keywords:** heat exchanger, crystallization fouling, electromagnetic field, heat transfer enhancement

## Abstract

In this study, the influence of an axial-electromagnetic field treatment device (AEFTD) with a solenoid structure using different electromagnetic frequencies on calcium carbonate (CaCO_3_) crystallization fouling on the tube side of a shell-and-tube heat exchanger was investigated. The experimental results indicated that the application of the AEFTD could effectively reduce fouling resistance and decelerate the growth rate of CaCO_3_ fouling. The opposite trend between fouling resistance and the outlet temperature of an experimental fluid indicated that the application of the AEFTD could enhance heat transfer. Meanwhile, the crystal morphologies of the fouling samples were analyzed by means of scanning electron microscopy (SEM). The axial-electromagnetic field favored the formation of vaterite as opposed to calcite. Non-adhesive vaterite did not easily aggregate into clusters and was suspended in bulk to form muddy fouling that could be carried away by turbulent flow. Furthermore, the anti-fouling mechanism of the axial-electromagnetic field is discussed in detail. The anti-fouling effect of the AEFTD on CaCO_3_ fouling exhibited extreme characteristics in this study. Therefore, the effectiveness of the AEFTD is contingent upon the selection of the electromagnetic parameters.

## 1. Introduction

Recycling cooling water is rich in Ca^2+^ and carbonic species. Many carbonates exhibit reverse solubility characteristics and gradually become oversaturated, precipitated, and accumulate on the heat exchange surface as the temperature increases, thus forming crystallization fouling, such as with calcium carbonate (CaCO_3_). Therefore, fouling formation is a common problem encountered in heat exchangers. The main concern with fouling is that it reduces heat transfer efficiency, affecting device life and production [1,2,3]. The increasing cost of energy and the decline in fossil fuels have intensified the emphasis on enhancing energy conversion efficiency. Therefore, the negative effects brought by fouling must be taken into account in the heat exchange field. Chemical and physical methods are the main approaches for controlling fouling in industrial production. Chemical methods include ion exchange, the preprecipitation of slightly soluble and insoluble salts, as well as the addition of fouling inhibitors [4,5]. However, these methods not only incur high costs but also change the physicochemical properties of aqueous solutions, potentially posing risks to human health and aquatic organisms. Chemical fouling inhibitors are highly effective in solving the fouling problem, yet they may produce synergistic effects and contain phosphate compounds that could lead to environmental harm, such as water eutrophication and algal bloom. Physical methods mainly encompass the application of electromagnetic fields [6], ultrasonic waves [7], catalytic materials [8], and other physical technologies. They induce alterations in the physical and chemical properties of aqueous solutions, leading to a reduction in salt supersaturation and fouling generation on heat exchange surfaces, so there will be no negative effects brought about by chemical methods. For example, the application of ultrasonic technology can induce significant cavitation and thermal effects in aqueous solutions, generating strong shock waves and microflows that prevent fouling [9,10]. Small amounts of catalytic materials, such as Zn, can decelerate the nucleation rate of CaCO_3_ and facilitate CaCO_3_ crystallization in the form of aragonite instead of calcite. This is a contributing factor in inhibiting fouling formation [11,12]. Among them, the electromagnetic treatment device, which generates an electromagnetic field inside the cavity through pulse signals of varying frequencies and amplitudes, is capable of treating aqueous solutions as they pass through. This treatment alters the physicochemical properties of the solution and modifies the CaCO_3_ crystallization process to achieve anti-fouling effects.

Previous studies have reported the application of electromagnetic fields in mitigating fouling and yielded favorable outcomes [13,14,15,16]. Simonič et al. [17] studied the impact of an electromagnetic field on the fouling behavior of pump diffusers in drinking water systems and analyzed the fouling components using scanning electron microscopy (SEM). The results revealed that in the experimental group, without the application of an electromagnetic field, CaCO_3_ precipitated as calcite; however, upon exposure to the electromagnetic field, it transformed into non-adhesive aragonite and could be easily removed by water flowing through the pump diffuser, thereby inhibiting fouling deposition. Additionally, it was found that the precipitation of aragonite required an electromagnetic treatment time of at least 2 h, which could be shortened under a higher magnetic induction intensity. Wang et al. [18] examined the influence of an electromagnetic field under different magnetic induction intensities (0, 0.01, and 0.02 T) on CaCO_3_ fouling in a shell-and-tube heat exchanger. The results showed that the asymptotic value of fouling resistance decreased as magnetic induction intensity increased, and the anti-fouling rates corresponding to 0.01 T and 0.02 T were 3.26% and 7.72%, respectively. Wang et al. [19] considered electromagnetic frequency as a crucial parameter in electromagnetic anti-fouling treatment and established a calculation model to determine the optimal electromagnetic frequency for inhibiting CaCO_3_ fouling in copper pipes under the action of an electromagnetic field. According to the calculation model, the ideal range for the electromagnetic frequency was found to be between 70–75 kHz. Then, the electromagnetic frequency range was partitioned into 0, 60–65, 65–70, 70–75, and 75–80 kHz for dynamic simulation experiments. The experimental results showed that the asymptotic value of fouling resistance initially decreased and then increased with increasing electromagnetic frequency, reaching its minimum within the range of 70–75 kHz. Under this working condition, the asymptotic value of fouling resistance decreased by 69.5% when compared to the untreated group. Kamar et al. [20] studied the influence of a low-frequency electromagnetic field on fouling in a plate heat exchanger. The results indicated that, when compared to the untreated group, the amount of fouling deposition on the heat exchange surface decreased by 80% after electromagnetic treatment. Most of the precipitates were calcite, and their average diameter reduced significantly. Zhang et al. [21] designed orthogonal experiments to discuss the synergistic effects of electromagnetic, flow, and temperature fields on CaCO_3_ fouling growth on the surface of a shell-and-tube heat exchanger. The experimental results indicated that the fouling resistance of a heat exchange surface decreased gradually with increasing magnetic induction intensity. The anti-fouling effect could be enhanced when the magnetic induction intensity and flow velocity acted together. Moreover, when the inlet temperature was 30 °C, the flow rate was 0.4 m/s, and the magnetic induction intensity was 20 mT, and the best experimental condition for anti-fouling effect was obtained in the paper.

The process of fouling formation is influenced by various factors, such as pH, temperature, concentration, and pressure, among others. Furthermore, fouling exhibits unique characteristics when subjected to electromagnetic fields. There is a dearth of widely accepted mechanisms to account for this experimental phenomenon. Despite the fact that the first patent was registered in the mid-1950s, the anti-fouling effect derived from electromagnetic fields remains a subject of numerous debates. Investigating fouling formation on heat transfer surfaces using electromagnetic fields and analyzing the relevant mechanism is helpful in further applying physical anti-fouling technology so as to achieve energy saving and consumption reductions. In this study, the primary objective was to investigate the influence of an axial-electromagnetic field treatment device (AEFTD) with a solenoid structure under different electromagnetic frequencies on CaCO_3_ crystallization fouling on the tube side of a shell-and-tube heat exchanger.

## 2. Materials and Methods

### 2.1. Experimental Rig

**Figure 1:**
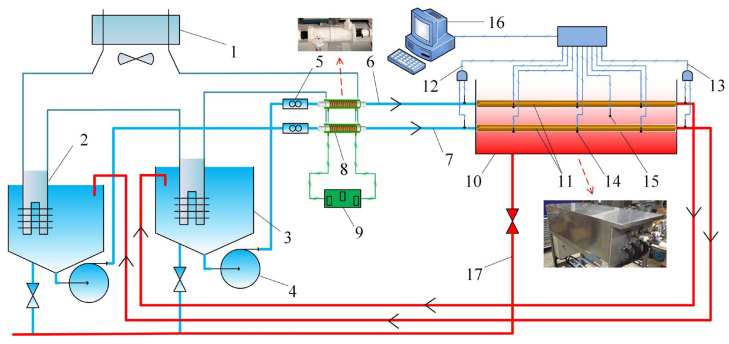
Schematic of the experimental rig. 1—Water chiller; 2—Cooling apparatus; 3—Experimental fluid tank; 4—Variable-frequency pump; 5—Ultrasonic flowmeter; 6—Circulation loop of side A; 7—Circulation loop of side B; 8—AEFTD; 9—Electromagnetic signal generator; 10—Thermostatic bath; 11—Shell-and-tube heat exchanger; 12—Inlet temperature; 13—Outlet temperature; 14—Wall temperature; 15—Bath temperature; 16—Data-acquisition system; 17—Drainage system.

Figure 1 shows a schematic of the experimental rig used in this study. It has two circulation loops with the same structure. The experimental fluid is pumped out from the experimental fluid tank by the variable-frequency pump, then passes through the ultrasonic flowmeter, AEFTD, and the experimental section, and returns to the experimental fluid tank. The variable-frequency pump can maintain the desired volume flow rate, which can be accurately measured using an ultrasonic flowmeter. The inlet temperature was kept constant through a water-cooling system, including a water chiller and cooling apparatus. The medium in the thermostatic bath is deionized water, for which the temperature was kept at a desired value by using five internal cartridge heaters. The temperatures of the experimental fluid at the inlet and outlet of the heat exchanger ($T_{\mathrm{in}}$ and $T_{\mathrm{out}}$) were measured by two DS18B20 digital-temperature sensors, respectively. The wall temperatures were measured using three DS18B20 digital temperature sensors uniformly installed under the shell side of the heat exchanger ($T_{w1}$ to $T_{w3}$). The temperature of thermostatic bath was measured by using a DS18B20 digital-temperature sensor inside the water. The temperature and volume flow rate data were uploaded to a data-acquisition system. All the temperature sensors were calibrated using a thermostat before the experiment.

**Figure 2:**
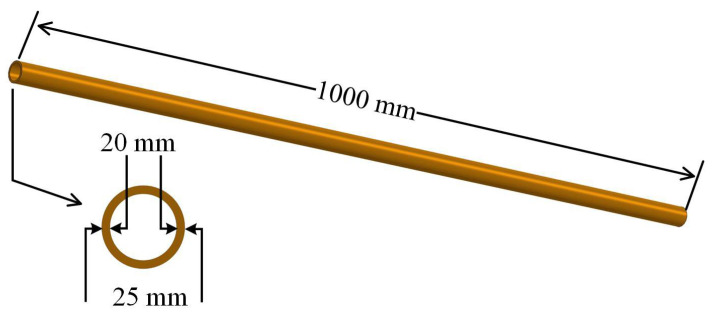
The shell-and-tube heat exchanger.

Figure 2 shows the shell-and-tube heat exchanger, made of red copper, with an effective length of 1000 mm for heat transfer. The internal and external diameters were 20 and 25 mm, respectively. The behavior of heat transfer will result in crystallization fouling formation on the tube side of the heat exchanger. The AEFTD is composed of a PVC tube, solenoid, and a cold-water shell. The solenoid is made of 21 layers of enameled copper wire entwined around the PVC tube. A voltage signal in the form of an alternating square wave with a specified amplitude and frequency is applied to the signal terminals of the solenoid, resulting in the generation of an axial-electromagnetic field within the treatment device. Due to the presence of resonant capacitors corresponding to different frequencies in the electromagnetic signal generator, a constant magnetic induction intensity can be maintained despite the change in frequency. During the experiment, the AEFTD generates a large amount of heat during operation. In order to prevent it from overheating, the cold water in the water chiller is pumped into the cold-water shell to absorb the heat from the solenoid and then flows back to the water chiller.

### 2.2. Experimental Process

The fouling generated in the previous experiment was removed using dilute hydrochloric acid before a new experiment was performed. Subsequently, the experimental rig was then repeatedly cleaned by using distilled water to prevent any residual acid. The steps for running the experiment were as follows:

(1) Distilled water (25 L) was introduced into two tanks. The electrical heater inside the thermostatic bath, variable-frequency pump, and water-cooling system was activated;
(2) When the experimental rig had been running for some time and reached a stable state, the volume flow rate was kept at 0.3 m^3^/h, the inlet temperature was kept at 30 °C, and the temperature of the thermostatic bath was kept at 60 °C. The temperature difference between the tube side and the shell side of the heat exchanger was 30 °C;
(3) The Na_2_CO_3_ and CaCl_2_ analytical reagents were separately dissolved in two measuring cups, each of which contained 1 L of distilled water from the experimental fluid tank, with a molar ratio of 1:1. Thereafter, the CaCl_2_ solution was first poured into the experimental fluid tank. After circulating for 5 min, the Na_2_CO_3_ solution was poured into the tank;
(4) Turning on the data acquisition system and electromagnetic signal generation: the experiment was considered complete when fouling resistance no longer increased. Both the circulation loops were subjected to the same operating procedure.

The specific working parameters are shown in Table 1.

Table 2 lists six experimental groups subjected to varying electromagnetic frequencies. Experiment-0 served as a control group without the application of AEFTD. In order to ensure a consistent magnetic induction intensity, the experimental groups using the AEFTD were subjected to a voltage of 40 V. This was carried out in order to investigate the impact of electromagnetic frequency on CaCO_3_ fouling on the tube side of heat exchangers.

### 2.3. Data Reduction

Fouling formation on the tube side of the heat exchanger increases heat-transfer resistance, which is termed fouling resistance. In this study, fouling resistance can be calculated by the following equation [22]:

(1) $$R_{f}=\frac{T_{w}-T_{s}}{q}$$

where $R_{f}$ is the fouling resistance (m^2^·K/W), $T_{w}$ is the wall temperature (°C), $T_{s}$ is the interface temperature (°C), and *q* is the heat flux (W/m^2^).

$T_{w}$ is the average wall temperature measured by three DS18B20 digital temperature sensors and can be calculated by the following equation:

(2) $$T_{w}=\frac{T_{\mathrm{wf}1}+T_{\mathrm{wf}2}+T_{\mathrm{wf}3}}{3}$$

where $T_{\mathrm{wf}1}$, $T_{\mathrm{wf}2}$, and $T_{\mathrm{wf}3}$ are the wall temperatures on the same heat exchanger (°C).

$T_{s}$ can be calculated by the following equation:

(3) $$T_{s}=\frac{T_{\mathrm{fo}}-e^{-\frac{4l}{d}St}T_{\mathrm{fi}}}{1-e^{-\frac{4l}{d}St}}$$

where $T_{\mathrm{fi}}$ and $T_{\mathrm{fo}}$ are the temperatures of the inlet and outlet (°C), respectively; *l* and *d* are the length and internal diameter of the heat exchanger (m), respectively; and St is the Stanton number.

*q* can be calculated by the following equation:

(4) $$q=\frac{Gc_{p}\rho (T_{\mathrm{fo}}-T_{\mathrm{fi}})}{\pi (d-2\delta_{f})l}$$

where $c_{p}$ and ρ are the specific-heat capacity (J/(kg·K)) and density (kg/m^3^) of the experimental fluid, respectively, and G is the volume flow rate of the experimental fluid (m^3^/h).

$\delta_{f}$ is the thickness of the fouling layer, which can be calculated by the following equation:

(5) $$\delta_{f}=0.5(1-\frac{\Delta p_{1}}{\Delta p_{2}})$$

where $\Delta p_{1}$ and $\Delta p_{2}$ are the pressure drops (Pa) before and after the $\delta_{f}$ change (m), respectively. In the calculation process, because $\delta_{f}$ is much smaller than *d*, $\delta_{f}$ can be considered negligible; that is, $\delta_{f}=0$.

The anti-fouling rate can be calculated by the following equation:

(6) $$\zeta =\frac{R_{f0}-R_{\mathrm{fm}}}{R_{f0}}\times 100\%$$

where ζ is the anti-fouling rate, $R_{f0}$ is the asymptotic value of fouling resistance without the AEFTD (m^2^·K/W), and $R_{\mathrm{fm}}$ is the asymptotic value of fouling resistance with the AEFTD (m^2^·K/W).

### 2.4. Uncertainty Analysis

The uncertainty of fouling resistance in this study can be calculated by the following equation:

(7) $$\mu_{R_{f}}=\frac{\partial R_{f}}{\partial T_{\mathrm{wf}1}}\mu_{T}^{\prime }+\frac{\partial R_{f}}{\partial T_{\mathrm{wf}2}}\mu_{T}^{\prime }+\frac{\partial R_{f}}{\partial T_{\mathrm{wf}3}}\mu_{T}^{\prime }+\frac{\partial R_{f}}{\partial T_{\mathrm{fi}}}\mu_{T}^{\prime }+\frac{\partial R_{f}}{\partial T_{\mathrm{fo}}}\mu_{T}^{\prime }+\frac{\partial R_{f}}{\partial G}\mu_{G}^{\prime }$$

where $\mu_{R_{f}}$ is the uncertainty of fouling resistance (m^2^·K/W); $\mu_{T}^{\prime }$ and $\mu_{G}^{\prime }$ are the compound uncertainties of temperature and volume flow rate, respectively; $\frac{\partial R_{f}}{\partial T_{\mathrm{wf}1}}$, $\frac{\partial R_{f}}{\partial T_{\mathrm{wf}2}}$, $\frac{\partial R_{f}}{\partial T_{\mathrm{wf}3}}$, $\frac{\partial R_{f}}{\partial T_{\mathrm{fi}}}$, $\frac{\partial R_{f}}{\partial T_{\mathrm{fo}}}$, and $\frac{\partial R_{f}}{\partial G}$ are transfer coefficients, which can be obtained from Equation (1).

$\mu_{T}^{\prime }$ can be calculated by the following equation:

(8) $$\mu_{T}^{\prime }=\sqrt{\mu_{T}^{2}+\mu_{\mathrm{CT}}^{2}}$$

where $\mu_{T}$ is the uncertainty of the DS18B20 temperature sensor (°C), and $\mu_{\mathrm{CT}}$ is the uncertainty of the intelligence data collection block (IDCB) during temperature measurement (°C).

$\mu_{G}^{\prime }$ can be obtained as follows:

(9) $$\mu_{G}^{\prime }=\sqrt{\mu_{G}^{2}+\mu_{\mathrm{CG}}^{2}}$$

where $\mu_{G}$ is the uncertainty of the ultrasonic flowmeter (m^3^/h), and $\mu_{\mathrm{CG}}$ is the uncertainty of the IDCB during the measurement of the volume flow rate (m^3^/h).

According to the operating instructions of the DS18B20 temperature sensor, its uncertainty is 0.167 °C [23], namely $\mu_{T}$.

The measurement error of the IDCB was ±(0.02% × measured value + 0.02% × measurement range) °C, and the temperature did not exceed 40 °C under all working conditions. The uncertainty of the IDCB during the temperature measurement is

(10) $$\mu_{\mathrm{CT}}=0.02\%\times 40+0.02\%\times 100=0.028\;{}^{\circ}C$$

The permissible error level of the ultrasonic flowmeter used in this study was 2%, and the flow range was 0.035–7 m^3^/h. Its uncertainty is

(11) $$\mu_{G}=2\%\times 7=0.14{\;m}^{3}/h$$

The volume flow rate was 0.3 m^3^/h as the experimental rig was running. The uncertainty of the IDCB during the volume flow rate measurement is

(12) $$\mu_{\mathrm{CG}}=0.02\%\times 0.3+0.02\%\times 7=0.00146{\;m}^{3}/h$$

Hence, $\mu_{R_{f}}$ is 4.812 × 10^−6^ m^2^·K/W.

## 3. Results and Discussion

### 3.1. Formation of Crystallization Fouling

**Figure 3:**
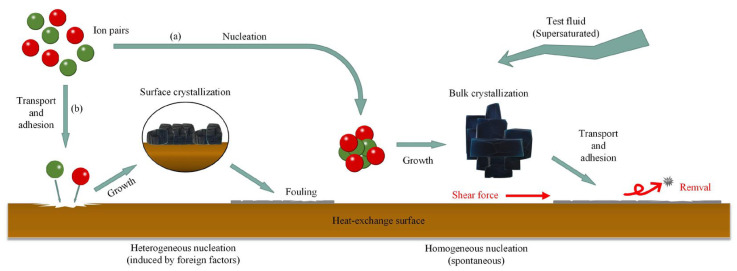
The formation process of crystallization fouling: (**a**): homogeneous precipitation; (**b**): heterogeneous precipitation.

Figure 3 shows the formation process of crystallization fouling on the heat exchange surface. A decrease in solution temperature will result in a reduction in solubility for salts with normal solubility. Due to supersaturation, the dissolved salt will crystallize and precipitate on the cooling surface. However, for salts with inverse solubility, the solubility decreases with increasing solution temperature, leading to supersaturation and the subsequent precipitation of the dissolved salts on heating surfaces. When the solution flows through the heat exchange surface, a boundary layer is generated with a temperature gradient. The CaCO_3_ dissolved in the solution is an insoluble salt with inverse solubility. It will be supersaturated at high temperatures, accelerate crystallization, and adsorb on the heat exchange surface, thus forming crystallization fouling, which can also be called water scale. In water-cooling systems, the most common fouling components are CaCO_3_, MgCO_3_, CaSO_4_, MgSO_4_, etc.

Although supersaturation is a necessary condition for the formation of crystallization fouling, it is not a sufficient condition. In fact, crystal growth requires a decrease in Gibbs free energy as the crystal size increases. If the crystal is smaller than its critical size, its Gibbs free energy will increase with increasing crystal size, and the crystal will tend to be redissolved in solution. Therefore, the solid boundary, such as the heat exchange surface, or suspended particles, like embryos, often serve as crystallization sites. As a result, there are two ways of forming crystallization fouling. The first way is that the ions, such as Ca^2+^ and CO_3_^2−^, form crystal nuclei in a solution and then grow into crystals (bulk crystallization). These crystal particles are then transported and attached to the heat exchange surface, which is called homogeneous precipitation, as shown in Figure 3a. The second way is that the ions are directly transported to the nearby heat exchange surface by the fluid. They make contact with the heat exchange surface and nucleate and grow into crystals, thus forming crystallization fouling (surface crystallization). This process is called heterogeneous precipitation, as shown in Figure 3b. During the process of heat transfer, both homogeneous and heterogeneous precipitation occur simultaneously. Therefore, the main factor affecting crystallization fouling is solution supersaturation. The higher the solution supersaturation, the stronger the driving force of crystallization. The heat exchanger surface is more likely to form crystallization fouling.

### 3.2. Fouling Resistance

**Figure 4:**
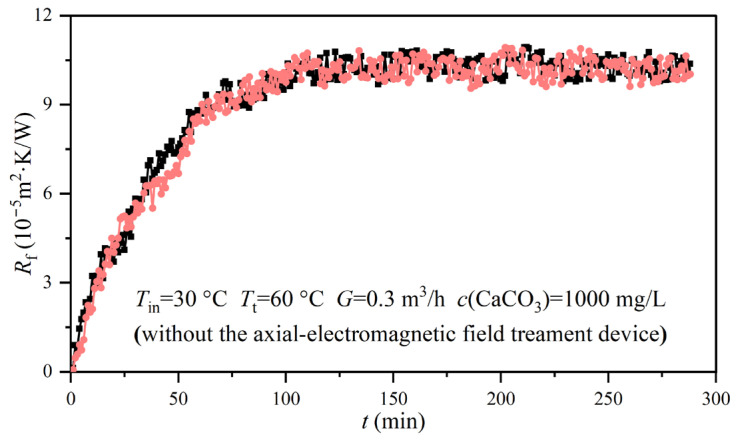
A comparison of the experimental results between the two groups of experiments.

In order to validate the accuracy of the data obtained from the experimental rig, two groups of experiments were performed under the same conditions. The inlet and thermostatic bath temperatures were 40 °C and 60 °C, respectively. The concentration of CaCO_3_ was 1000 mg/L, while the volume flow rate was 0.3 m^3^/h. No AEFTD was used to treat the experimental solution. A comparison of the experimental results between the two groups is presented in Figure 4. Fouling resistance was a function of time. The results showed that there was no significant difference between the two fouling resistance curves in the growth stage. After 150 min, the asymptotic values of fouling resistance tended to be consistent, with a discrepancy of only 3.23% between the two experimental groups, thus demonstrating the reproducibility of the results obtained from the experimental rig.

**Figure 5:**
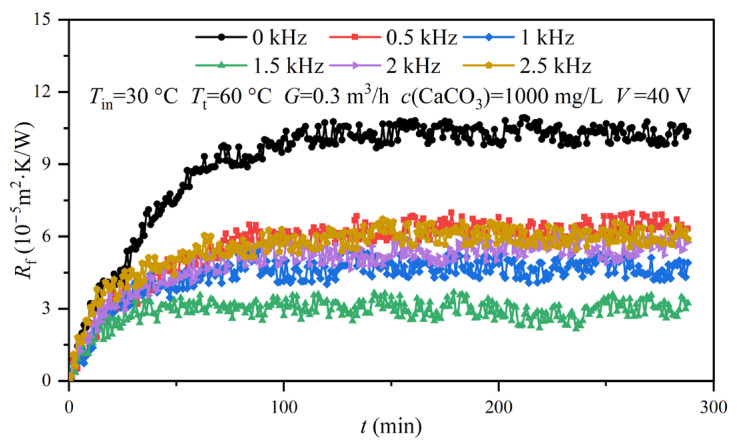
Fouling resistance curves under different electromagnetic frequencies.

Figure 5 shows six fouling resistance curves under different electromagnetic frequencies, while Table 1 and Table 2 present the specific experimental conditions. As the experimental fluid flowed through the heat exchange surface that was clean and without pollutants, the CaCO_3_ precipitated and gradually deposited on the tube side of the heat exchanger, thus resulting in fouling resistance. It can be observed from Figure 5 that, over the whole experimental period, the fouling resistance curves showed the same trend, rising first and then gradually stabilizing. Therefore, they can be divided into the rising stage and the stable stage. During the rising stage, the CaCO_3_ continually crystallized and adhered to the tube side [24]. The fouling resistance of the heat exchange surface rapidly increased, during which the deposition rate ($\varphi_{d}$) was higher than the removal rate ($\varphi_{r}$). Thereafter, as $\varphi_{d}$ was equivalent to $\varphi_{r}$, the fouling resistance reached the stable stage and ceased to increase. The fouling resistance curves show obvious differences under different electromagnetic frequencies. When compared to the control group, the fouling resistances with the AEFTD were greatly reduced, indicating that the application of the AEFTD could effectively inhibit CaCO_3_ fouling formation on the tube side of the heat exchanger. Xing [25] reached the same conclusion.

**Figure 6:**
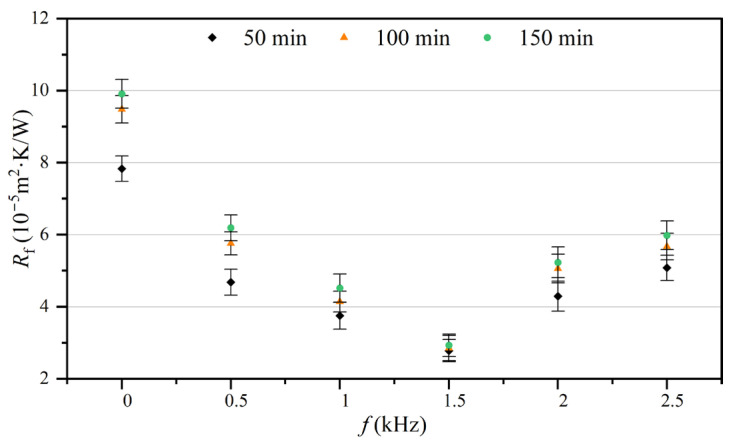
Fouling resistances at 50, 100, and 150 min under different electromagnetic frequencies.

Figure 6 shows the fouling resistances at 50, 100, and 150 min under different electromagnetic frequencies. By comparing the fouling resistances that have the same electromagnetic frequency at different moments, it can be found that the fouling resistance gradually increased as the experiment proceeded. However, different electromagnetic frequencies corresponded to varying degrees of fouling resistance increments. The fouling resistance increment in the control group was the highest, and 1.5 kHz had the smallest increment. In addition, at three specific time points, fouling resistance initially decreased and subsequently increased and reached its minimum at 1.5 kHz with increasing electromagnetic frequency.

The asymptotic value of fouling resistance can serve as an indicator for the final state of fouling accumulation on the tube side of a heat exchanger when the experimental system is in equilibrium. Achieving a smaller asymptotic value for fouling resistance is a desirable result when using an AEFTD. Table 3 shows the asymptotic values for the fouling resistance and anti-fouling rates under different experimental groups. The maximum asymptotic value for fouling resistance in the control group was 10.15 × 10^−5^ m^2^·K/W. The anti-fouling rate was between 36.65% and 71.13%, corresponding to different electromagnetic frequencies. When the electromagnetic frequency was 1.5 kHz, the asymptotic value of fouling resistance reached the minimum, which was 2.93 × 10^−5^ m^2^·K/W, with an anti-fouling rate of 71.13%. It can be found that the treatment effects of the axial-electromagnetic field are different under different electromagnetic frequencies. The effectiveness of the AEFTD is contingent upon the selection of the appropriate electromagnetic parameters. This study revealed an extreme character in the treatment effect of an axial-electromagnetic field, with the optimal performance achieved at the electromagnetic frequency of 1.5 kHz.

### 3.3. Growth Rate of CaCO_3_ Fouling

It is conceivable that during the fouling formation period, the fouling components will be deposited on the heat exchange surface, leading to an increase in fouling resistance. However, there is a phenomenon through which the fouling components are removed by fluid, thus resulting in a reduction in fouling resistance. Therefore, changes in fouling resistance over time are influenced by both the deposition and removal factors. The fouling formation process can be described using the following differential equation:

(13) $$\frac{dm_{f}}{dt}={\dot{m}}_{d}-{\dot{m}}_{r}$$

where $m_{f}$ is the mass of fouling deposition per unit area (kg/m^2^), ${\dot{m}}_{d}$ is the deposition rate (kg/(m^2^·s)), and ${\dot{m}}_{r}$ is the removal rate (kg/(m^2^·s)).

Assuming that the characteristics of the fouling components and the thickness of the fouling layer are evenly distributed along the heat exchange surface, the fouling resistance can be defined as follows:

(14) $$R_{f}=m_{f}/(\rho_{f}\lambda_{f})$$

where $\rho_{f}$ is the density of the fouling components (kg/m^3^), and $\lambda_{f}$ is the coefficient of thermal conductivity (W/(m·K)).

The differential of Equation (14) can be obtained as follows:

(15) $$dm_{f}=\rho_{f}\lambda_{f}dR_{f}$$

Thereafter, Equation (15) is substituted into Equation (13), and the result is as follows:

(16) $$\frac{dR_{f}}{dt}=\frac{{\dot{m}}_{d}-{\dot{m}}_{r}}{\rho_{f}\lambda_{f}}=\varphi_{d}-\varphi_{r}$$

where $\varphi_{d}$ is the deposition rate (m·K/N), and $\varphi_{r}$ is the removal rate (m·K/N).

Equation (16) indicates that the growth rate of fouling resistance per unit area can be described as the difference between the deposition rate ($\varphi_{d}$) and removal rate ($\varphi_{r}$), which is termed the Kern–Seaton model [22].

**Figure 7:**
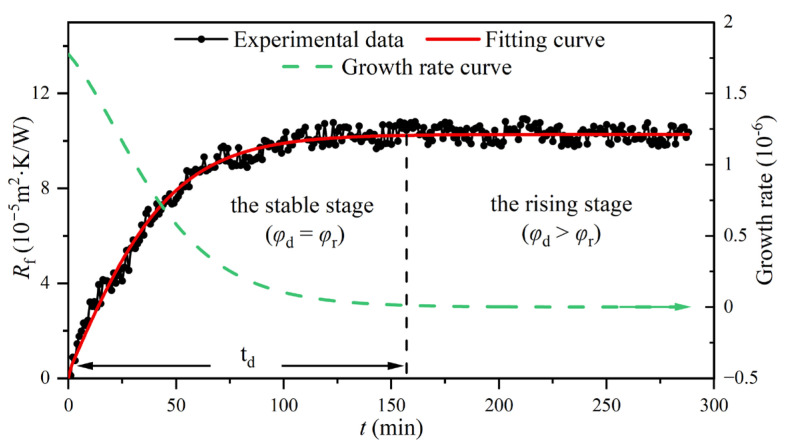
Fitting and growth rate curves of the fouling resistance in the control group.

As shown in Figure 7, by taking the experimental data from the control group as an example, it can be found that the entire period of the fouling resistance curve can be divided into two stages: the rising and stable stages. The experimental data were fitted using the Boltzmann function, and the R^2^ of the fitting curve marked in red was greater than 0.96, which indicated that the fitting effect was excellent and the fitting curve could correctly reflect the variation in the fouling resistance during the experiment. In addition, the R^2^s of the six fitting curves in the experimental groups were greater than 0.94.

The fitting curve marked in red in Figure 7 can be expressed as the following equation:

(17) $$R_{f}=A_{2}+(A_{1}-A_{2})/(1+\exp((t-t_{0})/A_{3}))$$

where $R_{f}$ is the fouling resistance (m^2^·K/W), *t* is the running time of the experimental rig (min), and *A*_1_ = −9.95 × 10^−5^, *A*_2_ = 1.03 × 10^−4^, *A*_3_ = 26.71, and *t*_0_ = −2.16.

The growth rate curve of fouling resistance, which is marked with a green dotted line, can be expressed by the differential equation of the fitting curve:

(18) $$\frac{dR_{f}}{dt}=(A_{2}-A_{1})\exp((t-t_{0})/A_{3})/A_{3}{\left(1+\exp((t-t_{0})/A_{3})\right)}^{2}$$

**Figure 8:**
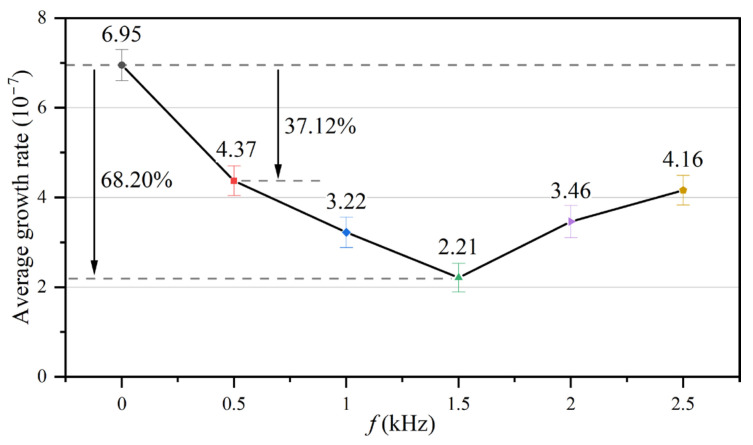
Average growth rates of CaCO_3_ fouling under different electromagnetic frequencies.

The fouling resistance of the heat exchange surface can characterize the accumulation state of CaCO_3_ fouling. Therefore, the growth rates of fouling resistance and CaCO_3_ fouling were considered equivalent. According to the above analysis, *t*_d_ in the control group (Figure 7) was taken as the deadline, and the average growth rates for CaCO_3_ fouling on the tube side of the heat exchanger under different electromagnetic frequencies were determined using Equation (18), as shown in Figure 8. The average growth rates showed a large difference with and without the AEFTD. When comparing the experimental results of the asymptotic value for the fouling resistances in Figure 6, both exhibit a similar trend with increasing electromagnetic frequency, indicating that the average growth rate was proportional to the asymptotic value of fouling resistance. In other words, the slower the growth rate, the smaller the fouling resistance asymptotic value. The average growth rate without the AEFTD was 6.95 × 10^−7^. After the application of the AEFTD, the average growth rates dropped by 37.12% to 68.20%, corresponding to different electromagnetic frequencies, indicating that CaCO_3_ fouling formation on the tube side was retarded. A minimum average growth rate of 2.21 × 10^−7^ was achieved at the electromagnetic frequency of 1.5 kHz. The reduced growth rate using the AEFTD is the desired result.

### 3.4. Outlet Temperature of Experimental Fluid

**Figure 9:**
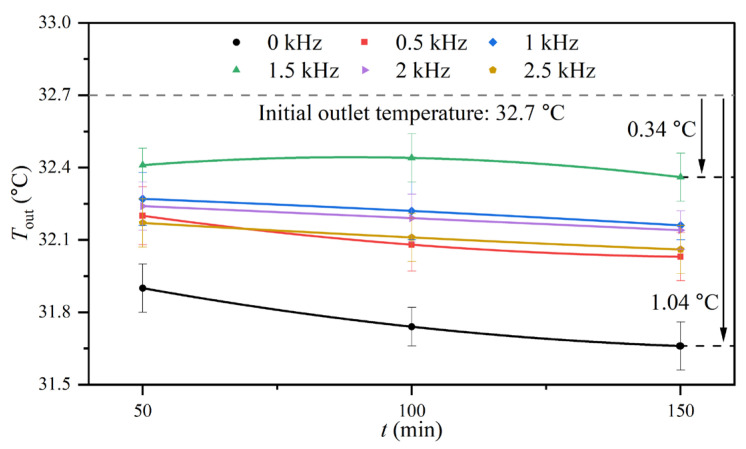
Outlet temperatures of experimental fluid at 50, 100, and 150 min under different electromagnetic frequencies.

During the experiment, the inlet temperature and volume flow rate of the experimental fluid and other working parameters remained constant. When the experimental fluid passed through the heat exchange surface, the heat exchange behavior resulted in an increase in the temperature of the experimental fluid. As the experiment proceeded, fouling accumulation on the heat exchange surface could reduce heat transfer efficiency, which led to a decrease in the outlet temperature of the experimental fluid. At the beginning of the experiment, the average outlet temperature of the experimental fluid was 32.7 ± 0.15 °C, indicating that the temperature of the experimental fluid had increased by 2.7 °C after passing through the shell-and-tube heat exchanger. Subsequently, the outlet temperature of the experimental fluid gradually dropped and then stabilized due to fouling. Figure 9 shows the outlet temperatures of the experimental fluid at 50, 100, and 150 min under different electromagnetic frequencies. The experimental results illustrated that the outlet temperatures of the experimental fluid with the AEFTD were higher than that of the control group. In addition, the outlet temperature of the experimental fluid at 1.5 kHz was significantly higher than that of the other experimental conditions. Except for the results of the control group and the 1.5 kHz condition, there was no significant difference in outlet temperatures among other experimental conditions.

Therefore, by combining the conclusions from Figure 5, the experimental results of the control group and 1.5 kHz condition were taken as examples for analysis. The asymptotic value of fouling resistance in the control group was the maximum: 10.15 × 10^−5^ m^2^·K/W, indicating that fouling accumulation on the heat exchange surface was more serious and obviously hindered heat transfer and the asymptotic value for fouling resistance at 1.5 kHz was the minimum: 2.93 × 10^−5^ m^2^·K/W. Meanwhile, it can be seen from Figure 9 that the outlet temperatures of the experimental fluid corresponding to the former were the lowest at each moment, while the outlet temperatures corresponding to the latter were the highest. When the experiment was carried out for 150 min and was compared with the initial outlet temperature, the outlet temperature of the control group decreased by 1.04 °C, and the outlet temperature of the 1.5 kHz condition decreased by 0.34 °C, indicating that the application of the AEFTD at 1.5 kHz increased the outlet temperature by 0.7 °C. The above analysis results show that the trend change was the opposite between fouling resistance and the outlet temperature of the experimental fluid, which indicated that the application of the AEFTD enhanced heat transfer.

### 3.5. SEM of CaCO_3_ Fouling

**Figure 10:**
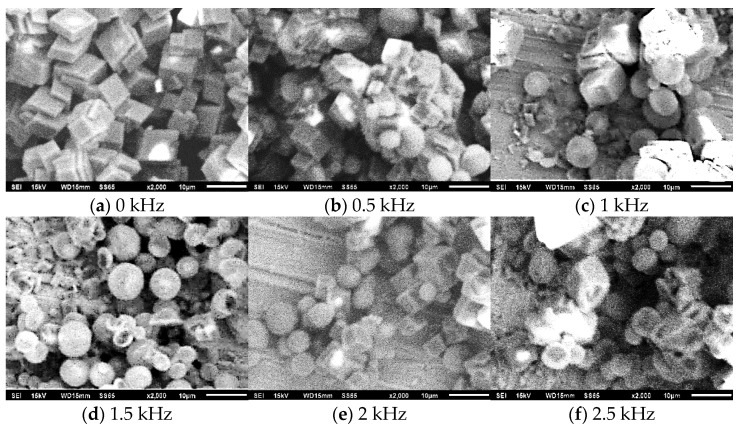
SEM micrographs of fouling samples under different electromagnetic frequencies.

Crystallization fouling is affinitive to the nucleation and growth of crystals. Therefore, variation in crystal morphology is also important for investigating the influence of the AEFTD on the crystallization fouling of a heat exchange surface. The three main types of CaCO_3_ crystal morphologies are vaterite, aragonite, and calcite [26], which belong to hexagonal, trigonal, and orthorhombic systems, respectively. Regarding the stability of the crystals, vaterite, with a spherical structure, is the least stable, followed by metastable aragonite with an acicular structure, whereas calcite, with a rhombohedral structure, forms the most stable crystal morphology and is widely found in nature. Prior to commencing the experiment, a red copper plate with a thickness of 0.5 mm, length of 20 mm, and width of 20 mm was bent and placed close to the tube side of the heat exchanger. The fouling samples on the red copper plate were dried and analyzed using SEM during the experiment. SEM micrographs (×2000) under different electromagnetic frequencies are shown in Figure 10.

According to the SEM micrographs in Figure 10, it can be found that the AEFTD has a significant impact on the morphology of CaCO_3_ crystals. The CaCO_3_ without the use of the AEFTD (Figure 10a) was found to be regular calcite with a rhombohedral structure and clustered together, which preferably formed dense hard fouling on the tube side of the heat exchanger and could not be easily removed. However, when compared to the control group, the morphology of the CaCO_3_ crystals was different from that of the axial-electromagnetic field. In addition to calcite, vaterite with a spherical structure was observed (Figure 10b–f). Vaterite is a nonpolar crystal, which does not easily aggregate into clusters and is suspended in the fluid to form muddy fouling that is carried away by turbulent flow, as opposed to accumulating on the tube side of the heat exchanger. Therefore, the appearance of vaterite crystals is a good phenomenon for inhibiting fouling formation on the tube side of the heat exchanger.

The proportion and size of vaterite in Figure 10b (0.5 kHz) were relatively small because the unstable crystal morphology grew further and recrystallized into calcite. However, with increasing electromagnetic frequency, the proportion of vaterite and crystal size increased. When the electromagnetic frequency was 1.5 kHz (Figure 10d), the CaCO_3_ remained vaterite in its morphology (with a large size) and did not transform into calcite. Vaterite was the major crystalline phase formed during this period. Furthermore, the proportion and size of vaterite slightly decreased when compared with those in Figure 10d (1.5 kHz), as the electromagnetic frequency continued to increase to 2.5 kHz (Figure 10f). These results indicate that the axial-electromagnetic field promoted CaCO_3_ crystallization in vaterite, as opposed to calcite. When combined with the experimental results obtained in Section 3.2, it was found that the formation of vaterite, which did not easily adhere to the tube side of the heat exchanger under the axial-electromagnetic field, was the main reason for the reduction in fouling on the heat exchange surface.

### 3.6. Anti-Fouling Mechanism

The anti-fouling mechanism of the AEFTD for crystallization fouling can be explained from two perspectives.

**Figure 11:**
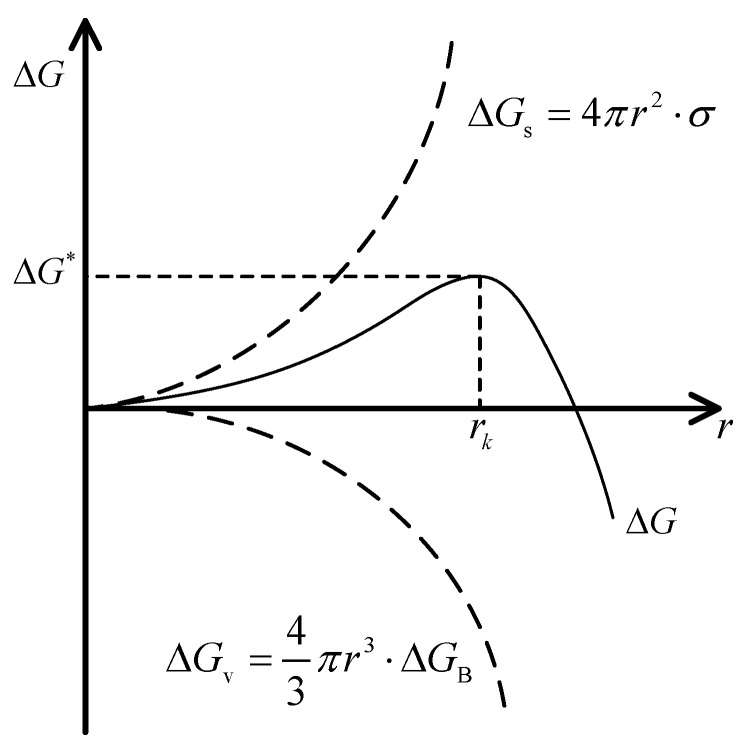
The relationship between the free energy variation and the radius of the crystal nucleus.

On the one hand, calcium carbonate is a kind of typical crystallization fouling. From the perspective of crystal growth, unstable vaterite was formed in the first stage through spheroidal crystal growth during CaCO_3_ crystal formation. This was followed by the conversion of vaterite to stable calcite in the second stage through dissolution and recrystallization. Crystal growth is accompanied by a decrease in the energy of the system, which is termed the Gibbs free energy [27]. Unstable crystalline phases first appeared and were gradually transformed into stable products. The system achieved minimum energy when the reaction reached equilibrium. Crystal nucleation is the first step of crystal growth, and its driving force is determined by the difference in volume-free energy between CaCO_3_ crystals and the experimental fluid. This force is proportional to the degree of supersaturation in the experimental fluid. The resistance to nucleation is the interfacial energy between the CaCO_3_ crystals and the experimental fluid. Therefore, there is a critical radius for the crystal nucleus, $r_{k}$. When $r>r_{k}$, the embryonic crystal can form a crystal nucleus. The relationship between the variation in free energy and the radius of a crystal nucleus is shown in Figure 11.

The free energy variation can be calculated as follows:

(19) $$\Delta G=-\Delta G_{V}+\Delta G_{S}$$

where ΔG is the free energy variation (J), $\Delta G_{V}$ is the volume of free-energy difference between the liquid and solid phases in the system (J), and $\Delta G_{S}$ is the interfacial energy in the system (J).

$\Delta G_{V}$ and $\Delta G_{S}$ can be calculated as follows:

(20) $$\Delta G_{S}=4\pi r^{2}\cdot \sigma$$

(21) $$\Delta G_{V}=\frac{4}{3}\pi r^{3}\cdot \Delta G_{B}$$

where $\Delta G_{B}$ is the free energy difference per unit volume (J/m^3^), and σ is the surface energy per unit area (J/m^2^), i.e., the specific surface energy.

When combined with the axial-electromagnetic field, there are many particles associated with fouling in the experimental fluid, such as with Ca^2+^, CO_3_^2−^, and CaCO_3_ crystals, and they have their own natural frequencies. When the electromagnetic frequency is close to or consistent with their natural frequencies, the particles resonate, absorbing more energy from the electromagnetic field, transforming this into internal energy in the fluid. Different frequencies correspond to different energies absorbed by the experimental fluid. When $0<r<r_{k}$, the energy provided by the electromagnetic field will increase ΔG so as to reach the nucleation barrier $\Delta G^{*}$ quickly and promote the growth of the embryonic crystal to the critical radius, *r*. When $r>r_{k}$, the volume-free energy becomes dominant, resulting in a decrease in ΔG, promoting further crystal growth. However, the energy from the electromagnetic field will inhibit the decrease in the ΔG, which can prevent the crystal size from continuing to grow and maintain the crystal in a metastable state. As a result, the decrease in Gibbs free energy was suppressed [28], thus impeding the transformation of vaterite into calcite. SEM micrographs of the fouling samples show the crystal morphology of vaterite. The greater the amount of energy absorbed by the fluid, the better the effect of inhibiting the transformation of vaterite to calcite. Fouling formation on the tube side of the heat exchanger surface was inhibited. In this study, the experimental fluid absorbed the most energy from the axial-electromagnetic field at 1.5 kHz.

On the other hand, water is the most common carrier of crystallization fouling in nature. Deionized water was used as the experimental fluid in this study. The angle between the two hydrogen-oxygen bonds in a water molecule is 104.5°. As a result, the center of oxygen and hydrogen atoms do not coincide, and water molecules are polar, which causes a type of electrostatic attraction force between the positive and negative charges of the two water molecules, namely the hydrogen bond. Due to the existence of hydrogen bonds, multiple individual water molecules will be associated with water molecular clusters [29]. Because the charges at each end of the hydrogen bond have opposite electrical properties. When the experimental fluid passes through the AEFTD, the Lorentz force from the axial-electromagnetic field will change some of the properties of the water body. The equation is shown as follows:

(22) $$\vec{F}=q\vec{v}\times \vec{B}$$

where *q* is the quantity of the charge (C), $\vec{v}$ is its velocity vector (m/s), and $\vec{B}$ is the magnetic induction intensity vector (T).

The mass center of the molecular water cluster is on the central oxygen atom, and the whole water molecular cluster moves around this oxygen atom, around which the whole cluster moves and rotates. The water molecular clusters are in equilibrium without the axial-alternating magnetic field, and under the action of the axial-alternating magnetic field, when the rotation axis of the water cluster is in line with the direction of the axial-alternating magnetic field, the water molecules will also rotate around the central axis. The energy of the hydrogen bond is very sensitive to the distance between the two charges. As can be seen from Equation (23), the axial-electromagnetic field causes positive and negative charges (linked by hydrogen bonds) to move in opposite directions periodically, which breaks or distorts the hydrogen bond, hence weakening or destroying the hydrogen bond network in the molecular water clusters. This results in the formation of smaller clusters, water molecule dimers, or individual water molecules.

The spectroscopy results also showed that the angle of the hydrogen bond in a water molecule decreased from 104.5° to 103.0° under the action of the magnetic field, indicating the distortion of the hydrogen bond [30]. Toledo et al. [31] obtained a similar conclusion through theoretical calculations, where a magnetic field weakened the hydrogen bonds between the water molecules and destroyed larger molecular water clusters, which formed more and smaller molecular water clusters with stronger hydrogen bonds.

According to the Stokes–Einstein equation [32], the self-diffusion coefficient of a particle is inversely proportional to its radius, which is defined as follows:

(23) $$d(H)=\frac{kT}{3\pi \eta D}$$

where d(H) is the radius of the particle (m), *k* is the Boltzmann constant (J/K), *T* is the thermodynamic temperature (K), η is the viscosity (Pa·s), and *D* is the self-diffusion coefficient (m^2^/s).

Therefore, the axial-electromagnetic field enhances the diffusivity and activity of water molecules. When combined with the experimental conditions in this study, the experimental fluid contains molecular water clusters, Ca^2+^, CO_3_^2−^, and CaCO_3_ crystals. Ca^2+^, CO_3_^2−^, and CaCO_3_ crystals have a layer of water molecules arranged in order. The former two are formed by hydration, which is called hydration ions. These peripheral water molecules provide a barrier to the interaction between Ca^2+^ and CO_3_^2−^ and to the adsorption of Ca^2+^ and CO_3_^2−^ on the surface of the CaCO_3_ crystals. Under the action of an axial-electromagnetic field of 1.5 kHz, the diffusion capacity of water molecules is significantly enhanced, and the peripheral water molecules will break free, thus promoting the reaction between Ca^2+^ and CO_3_^2−^ and the homogeneous nucleation of CaCO_3_ crystals in bulk, which reduces the probability of CaCO_3_ adhering to the tube side of the heat exchanger.

## 4. Conclusions

In this study, the influence of an AEFTD using different electromagnetic frequencies on calcium carbonate (CaCO_3_) crystallization fouling on the tube side of a shell-and-tube heat exchanger was investigated. The experimental results indicated that the application of the AEFTD could effectively reduce fouling resistance and decelerate the growth rate of CaCO_3_ fouling. The anti-fouling rate was between 36.65% and 71.13%, corresponding to different electromagnetic frequencies. The opposite trend between fouling resistance and the outlet temperature of the experimental fluid indicated that the application of the AEFTD could enhance heat transfer. Furthermore, the axial-electromagnetic field favored the formation of vaterite as opposed to calcite. Non-adhesive vaterite did not easily aggregate into clusters and was suspended in bulk to form muddy fouling that could be carried away by turbulent flow. The anti-fouling effect of the AEFTD on CaCO_3_ fouling exhibited extreme characteristics in this study. Therefore, the effectiveness of the AEFTD is contingent upon the selection of the electromagnetic parameters. Subsequent studies will explore the synergistic effect of electromagnetic frequency and magnetic induction intensity so as to obtain the optimal electromagnetic parameters for fouling treatment.

## Figures and Tables

**Table 1:** Working parameters.

Parameter	Specific Value
The volume of distilled water	25 L
Na_2_CO_3_ analytical reagent	26.5 g
CaCl_2_ analytical reagent	27.75 g
Hardness of experimental fluid	1000 mg/L
Inlet temperature	30 °C
Bath temperature	60 °C
Volume flow rate	0.3 m^3^/s

**Table 2:** Axial-electromagnetic field parameters of experimental groups.

Experimental Group	Frequency (kHz)	Voltage (V)
Experiment-0	0	0
Experiment-1	0.5	40
Experiment-2	1	40
Experiment-3	1.5	40
Experiment-4	2	40
Experiment-5	2.5	40

**Table 3:** Asymptotic values of fouling resistance and anti-fouling rates under different electromagnetic frequencies.

Experimental Group	Asymptotic Value of Fouling Resistance (10^−5^ m^2^·K/W)	Anti-Fouling Rate (%)
Experiment-0	10.15	None
Experiment-1	6.43	36.65
Experiment-2	4.64	54.29
Experiment-3	2.93	71.13
Experiment-4	5.59	44.93
Experiment-5	6.07	40.20
Experiment-0	10.15	None

## Data Availability

Data sharing is not applicable.

## Nomenclature

**Symbol**	**Description**	**Unit**
$R_{f}$	fouling resistance	m^2^·K/W
$T_{w}$	wall temperature	°C
$T_{s}$	interface temperature	°C
*q*	heat flux	W/m^2^
$T_{\mathrm{fi}}$	inlet temperature	°C
$T_{\mathrm{fo}}$	outlet temperature	°C
*l*	length of the heat exchanger	m
*d*	internal diameter of the heat exchanger	m
St	Stanton number	
$c_{p}$	specific-heat capacity	J/(kg·K)
ρ	density	kg/m^3^
G	volume flow rate	m^3^/h
$\delta_{f}$	thickness of the fouling layer	m
Δp	pressure	Pa
ζ	anti-fouling rate	
$R_{f0}$	asymptotic value of fouling resistance without the AEFTD	m^2^·K/W
$R_{\mathrm{fm}}$	asymptotic value of fouling resistance with the AEFTD	m^2^·K/W
$\mu_{R_{f}}$	uncertainty of fouling resistance	m^2^·K/W
$\mu_{T}^{\prime }$	compound uncertainties of temperature	°C
$\mu_{G}^{\prime }$	compound uncertainties of volume flow rate	m^3^/h
$m_{f}$	mass of fouling deposition per unit area	kg/m^2^
${\dot{m}}_{d}$	deposition rate	kg/(m^2^·s)
${\dot{m}}_{r}$	removal rate	kg/(m^2^·s)
$\rho_{f}$	density of the fouling components	kg/m^3^
$\lambda_{f}$	coefficient of thermal conductivity	W/(m·K)
$\varphi_{d}$	deposition rate	m·K/N
$\varphi_{r}$	removal rate	m·K/N
t	running time of the experimental rig	min
ΔG	free energy variation	J
$\Delta G_{V}$	volume free energy difference	J
$\Delta G_{S}$	interfacial energy	J
$\Delta G^{*}$	nucleation barrier	J
$\Delta G_{B}$	free energy difference per unit volume	J/m^3^
σ	surface energy per unit area	J/m^2^
q	quantity of charge	C
$\vec{v}$	velocity vector	m/s
$\vec{B}$	magnetic induction intensity	T
d(H)	radius of the particle	m
k	Boltzmann constant	J/K
T	thermodynamic temperature	K
η	viscosity	Pa·s
D	self-diffusion coefficient	m^2^/s
